# PKZ, a Fish-Unique eIF2α Kinase Involved in Innate Immune Response

**DOI:** 10.3389/fimmu.2020.00585

**Published:** 2020-03-31

**Authors:** Chuxin Wu, Yibing Zhang, Chengyu Hu

**Affiliations:** ^1^Department of Natural Sciences, Yuzhang Normal University, Nanchang, China; ^2^State Key Laboratory of Freshwater Ecology and Biotechnology, Institute of Hydrobiology, Chinese Academy of Sciences, Wuhan, China; ^3^Department of Bioscience, College of Life Sciences, Nanchang University, Nanchang, China

**Keywords:** PKZ, Zα domain, Z-DNA, kinase activity, fish

## Abstract

PKZ is a novel and unique eIF2α protein kinase identified in fish. Although PKZ is most homologous to PKR, particularly in the C-terminal catalytic domain, it contains two N-terminal Z-DNA-binding domains (Zα1 and Zα2) instead of the dsRNA binding domains (dsRBDs) in PKR. As a novel member of eIF2α kinase family, the available data suggest that PKZ has some distinct mechanisms for recognition, binding, and B-Z DNA transition. Functionally, PKZ seems to be activated by the binding of Zα to Z-DNA and participates in innate immune responses. In this review, we summarize the recent progress on fish PKZ.

## Introduction

In eukaryotes, translation initiation is an extremely complicated process, and regulation of mRNA translation is very important for a cell to adapt to various stress conditions. As an important mechanism for control of protein synthesis, phosphorylation, or dephosphorylation of eukaryotic translation initiation factor 2 α subunit (eIF2α) represents a core molecular switch for stress adaptation and rapid metabolic regulation (1). In mammals, eIF2α is phosphorylated at Ser51 by a family of four kinases, HRI (heme-regulated inhibitor), PERK (PKR-like endoplasmic reticulum kinase), GCN2 (general control non-derepressible-2), and PKR (double-stranded RNA-dependent protein kinase). HRI is a sensor of heme deprivation and arsenite exposure. PERK is primarily activated by endoplasmic reticulum stress. GCN2 is activated under conditions of amino acid and glucose deprivation. PKR is a well-known dsRNA-activated and interferon (IFN)-stimulated protein, and plays significant roles in antiviral immune response (2–4).

The past few decades have witnessed significant progress in understanding of fish innate immune, and four eIF2α kinases analogous to those in mammals have been identified in fish (5–13). Intriguingly, fish have some unique antiviral-relevant or immune-relevant genes such as PKZ, the fifth member of eIF2α kinase family. PKZ has a closer evolutionary relationship with PKR (14). Functionally, it protects fish cells against viral infection through phosphorylation of eIF2α and may act as a cytosolic DNA sensor to initiate innate immune response (15). In this review, we summarize the recent progress on fish PKZ and its role in innate immune response.

## Discovery of PKZ as a PKR-Like Protein Kinase

When fish cells are infected with the virus, many fish IFN-stimulated genes (ISGs) are induced, most of which are homologous to the known mammalian ISGs, and some of which are novel. Crucian carp *PKZ* (*CaPKZ*) is such a fish-specific gene, and was named *CaPKR-like* at that time when it was first identified from IFN-producing CAB cells after treatment with UV-inactivated GCRV in 2004 (16). The full-length cDNA of *CaPKZ* is 2192 bp with an ORF encoding a polypeptide of 513 amino acid residues. Although the protein size and C-terminal 11 catalytic sub-domains of *Ca*PKZ are most similar to that of mammalian PKR proteins, *Ca*PKZ catalytic domain possesses a large and variant insert (≈85 residues) between sub-domains IV and V, in contrast to a short insert (≈10–34 residues) in PKR. Specially, *Ca*PKZ has two Z-DNA binding domains (Zα1 and Zα2) within its N-terminus instead of typical dsRNA binding domains (dsRBDs) in PKR.

Since then, the homologous genes of *CaPKZ* have been cloned from Zebrafish (*Danio rerio*) (17), Atlantic salmon (*Salmo salar*) (18), Rare minnow (*Gobiocypris rarus*) (19) and Grass carp (*Ctenopharyngodon idellus*) (20), indicating there exists a novel eIF2α kinase exclusively in fish. In Zebrafish, *PKZ* gene is made up of 11 exons and transcribes four alternative splicing variants (A–D, named in order of decreasing abundance) (17, 21). Variant A codes the entire protein. Variant B codes a protein that lacks the kinase insert domain of 78 aa. Variants C and D encode truncated proteins because they retain an intron providing a premature stop codon (17).

## Similarities and Differences of PKZ and PKR

**FIGURE 1:**
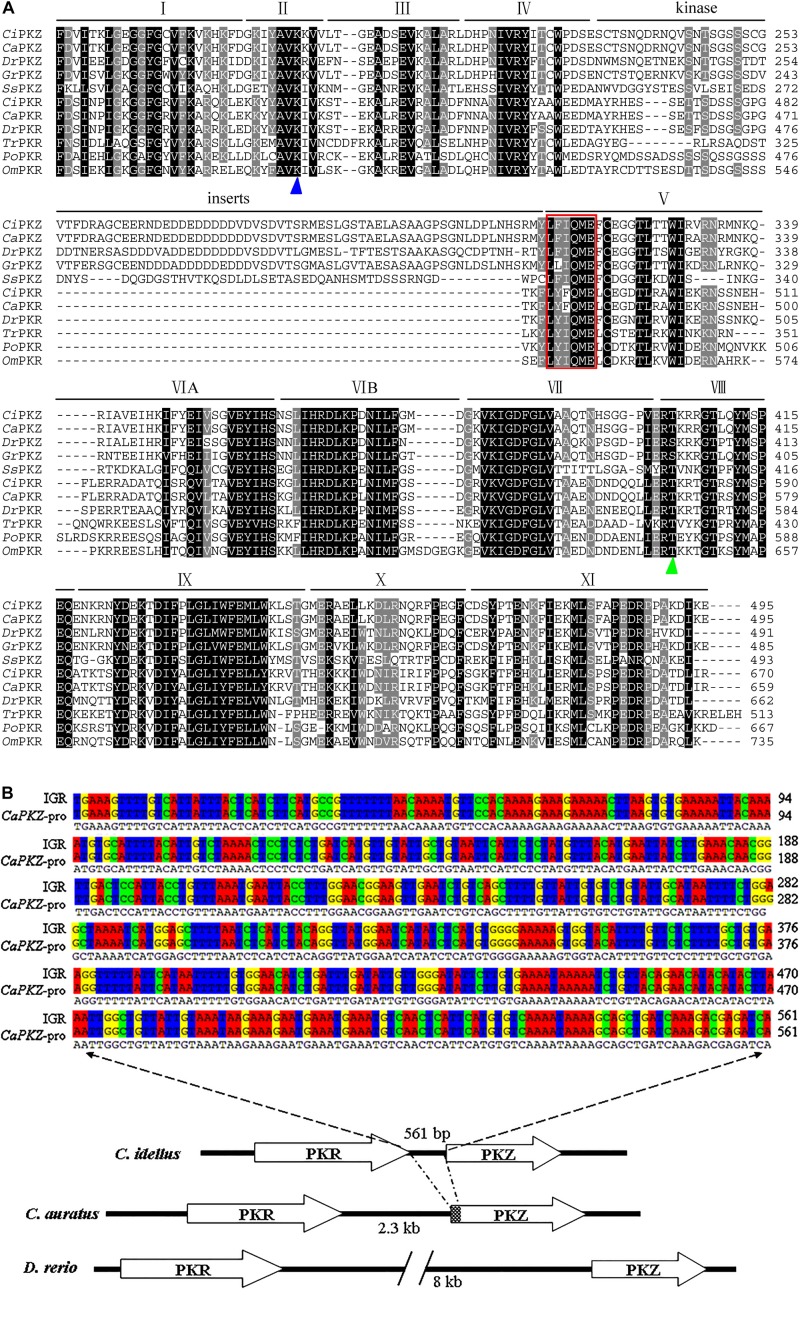
Multiple sequence alignment and genomic arrangement of fish *PKR* and *PKZ* genes. **(A)** Multiple sequence alignment of the kinase domains of fish PKR and PKZ by Clustal X 2.0 program. Residues Lysine (K) for PKR/PKZ catalytic activity (blue triangle) and Serine/Threonine for autophosphorylation (green triangle) are marked under the sequences. The conserved sequence of LFIQME(Y/F)C(D/E) in subdomain V is surrounded by a red box. Identical (shaded in black) and similar (shaded in gray and light gray) residues are indicated. The following abbreviations were used: *Ci*, *Ctenopharyngodon idellus*; *Ca*, *Carassius auratus*; *Dr*, *Danio rerio*; *Ss*, *Salmo salar*; *Gr*, *Gobiocypris rarus*; *Tr*, *Takifugu rubripes*; *Po*, *Paralichthys olivaceus*; *Om*, *Oncorhynchus mykiss*. **(B)** Genomic arrangement of tandemly arranged known fish *PKR* and *PKZ* genes. The genomic arrangement and relative orientation of PKR and PKZ genes in *C. idellus*, *C. auratus*, and *D. rerio* are shown. Arrows indicate the 5’ to 3’ orientation of the genes. The approximate sizes of intergenic regions are indicated below. Two sequence alignment of the intergenic region (IGR) between *CiPKR* and *CiPKZ* and the partial sequence of *Ca*PKZ promoter are displayed above.

Structurally, PKZ resembles PKR, containing an N-terminal regulatory domain and a C-terminal eIF2α kinase domain. Also, the kinase domain of fish PKZ is around 65.42% identical to that of fish PKR (Figure 1A). The striking difference between PKZ and PKR lies in the kinase insert (KI) linking kinase subdomains IV and V, which is necessary for phosphorylation of eIF2α (21). As for the N-terminal regulatory region, it is highly divergent. PKZ has two Zα domains, while PKR has dsRBDs in its N-terminus. It is noted that unlike mammalian and amphibian PKRs possessing two dsRBDs, fish PKRs own one, two or three dsRBDs (21).

Similar to the genomic arrangement of the three *X. tropicalis PKR* genes, fish PKR, and PKZ genes are tandemly arranged in head-to-tail (parallel) orientation in the genome of zebrafish, crucian carp or grass carp (21, 22). However, searching the Grass Carp Genome Database (GCGD) shows that the length of intergenic region (IGR) between *PKZ* and *PKR* in grass carp genome is very short, only 561 bp, which is different from that of zebrafish or crucian carp. Interestingly, the nucleotide sequence of the IGR and the partial sequence of *CaPKZ* promoter show 99.8% (560/561) sequence identity (Figure 1B). Whether the size divergence of IGR is related to the intensity and rapidity of expression and regulation of *PKZ* gene in different species is not known. Phylogenetic analysis shows that the kinase domains of fish PKRs are more closely related to those of fish PKZs than to non-fish vertebrate PKRs. It is speculated that a gene duplication event generating fish *PKR* and *PKZ* genes occurred early during teleost fish evolution after the divergence of the tetrapod lineage, and the Zα domains replaced the dsRBDs in fish (21).

Since PKZ and PKR coexist in fish as neighboring genes, their functions in cells may be overlapping and distinct. In CAB cells, both PKZ and PKR are activated by IFN or some IFN stimuli resulting in phosphorylation of eIF2α and inhibition of virus replication. The effect of both kinases together is much more significant than either of them, and PKZ seems to exhibit a weaker antiviral ability than PKR, correlating with its lower ability to phosphorylate eIF2α than PKR. Moreover, the activation patterns and functions of PKZ and PKR in cells vary greatly (22).

## Function Domains of PKZ

### Zα Domains and Its Interaction With Z-DNA

Zα domains belong to a subfamily of wHTH domains with the unique property of specific binding to Z-DNA (23). The highly conserved Zα domain is observed in human ADAR1 (24), DAI (25), vaccinia virus E3L protein (26), and PKZ. Typically, the Zα domain contains three α-helices and three antiparallel β-strands arranged in α1β1α2α3β2β3 topology. Helix 3 and the β-loop between strands β2 and β3 have been found to interact with DNA (27). The β-loop participates in Z-DNA binding in conjunction with the recognition helix 3 and forms the “wing” structure. The conserved proline residues in the wing make hydrophobic contacts with the phosphate backbone of Z-DNA (23, 28).

Current structural analyses imply that the Zα domain of PKZ (Zα_PKZ_) has distinguished features from other Zα domains. There is an extended loop between β2 and β3 strands of *Dr*Zα_PKZ_, forming the largest β-wing in the known Zα domains (29). The Arg62 residue of the extended wing of *Dr*Zα_PKZ_ leads to interact with the primary DNA strand and lost for binding at the edge of the DNA duplex (30). As for *Ca*PKZ Zα (*Ca*Zα_PKZ_), it recognizes P0–P4 phosphates of Z-DNA, while other Zα domains interact with P1–P5 phosphates (31).

As a novel protein containing the Zα domain, fish PKZ Zα exhibits a high binding potential to Z-DNA (17, 18, 32–34), and the sub-domains Zα1 and Zα2 undertake diverse functions, showing that Zα1 is more efficient than Zα2 in B–Z conformational transition (32, 33). Zα_PKZ_ can recognize and convert B-DNA or B-RNA to Z-conformation, which serves as a “flippase” similar to Zα_*ADAR1*_ (33, 35, 36). Several conserved residues in Zα are essential for Z-DNA recognition and binding. In Zα_*ADAR1*_, nine residues (K169, K170, N173, R174, Y177, T191, P192, P193, and W195) are important for Z-DNA recognition and binding (25, 27). Similarly, the subdomain Zα1 of PKZ contains nine residues as Zα_*ADAR1*_. These conserved residues in Zα_PKZ_ play a critical role in the B–Z transition of DNA (33).

Intriguingly, the B-Z transition activity of Zα_PKZ_ is strongly salt concentration-dependent, unlike other Zα proteins such as *h*Zα_*ADAR1*_ and *Yab*Zα_E3L_. With increasing of [NaCl] from 10 to 250 mM, the B-Z transition activity of *Ca*Zα_PKZ_ is impaired severely (37). Similarly, *Dr*Zα_PKZ_ has the ability to convert poly(dG-dC) into the Z-DNA in the presence of low amounts of cobalt hexamine (17). In addition, *Dr*Zα_PKZ_ has two positively charged residues (Lys61 and Arg62) in the extended β-wing involved in B–Z DNA transition, whereas *Ca*Zα_PKZ_ has only one positively charged residue (Lys56). It may be a reason why the B–Z transition induced by *Dr*Zα_PKZ_ is faster than by *Ca*Zα_PKZ_ (29).

### Kinase Domains and eIF2α Kinase Activity of PKZ

Gene organization of *CiPKZ* and *DrPKZ* reveals that the kinase domain is encoded by seven exons, and the kinase insert is encoded by four exons. A distinguishing sequence of LFIQME(Y/F)C(D/E) in subdomain V of PKZ is proposed to be important for eIF2α kinase activity (16, 17) (Figure 1A). Sequence alignment and site-directed mutagenesis studies indicate that a conserved Lys is important for catalytic activity, located at position 199 in *Dr*PKZ, 198 in *Ca*PKZ or *Ci*PKZ. At position 402 in *Dr*PKZ is a Ser (corresponding to Thr-404 in *Ca*PKZ or *Ci*PKZ), allowing for autophosphorylation (17, 20) (Figure 1A).

In mammals, PKR functions as an eIF2α kinase. EIF2α can be phosphorylated at Ser51 by PKR, leading to inhibition of general protein synthesis and initiation of apoptosis (38, 39). Given a highly homologous kinase region, fish PKZ has similar function features as PKR. *In vitro*, recombinant PKZ autophosphorylates and phosphorylates eIF2α in the absence of any activators. The dephosphorylated PKZ is activated again by poly(dG-dC) but not poly(I:C). PKZ shows an ability to phosphorylate recombinant wild-type eIF2α but not the recombinant non-phosphorylatable variant eIF2α (S51A). Furthermore, the wild-type PKZ, but not the kinase defective variant (K198R in *Ca*PKZ/*Ci*PKZ, K199R in *Dr*PKZ, and K217R in *Ss*PKZ), exhibits a direct inhibitory effect on reporter gene expression (17, 18, 20, 22). In addition, both of two *Dr*PKZ isoforms (*Dr*PKZ-A and *Dr*PKZ-B) functionally interact with eIF2α and inhibit protein synthesis *in vivo*. Deletion of the insert domain of *Dr*PKZ-A or *Dr*PKZ-B results in abrogating the kinase activity completely. It indicates that the insert domain is required for *Dr*PKZ kinase activity. Kinase activity appears to be independent of the insert length, while it depends on the presence of specific amino acids within the insert domain (40).

## Roles of PKZ in Fish Innate Immune Responses

### Being Induced as a Typical ISG

It is well known that IFN exerts antiviral effects through induction of hundreds of ISGs, including PKR, Mx1, ADAR1, ISG15, viperin, and so on (14). Coexistence of *PKR* and *PKZ* in a head-to-tail orientation in fish genomes is believed to be very important for similar transcriptional activation after immunostimulation. Actually, *PKZ* is up-regulated by virus infection and lots of IFN stimuli, including IFN, poly(I:C), poly(dA-dT), poly(dG-dC), genomic DNA, and even *Aeromonas hydrophila* (16–22, 40). poly(I:C)-induced of PKZ requires novel protein synthesis and IFN stimulates PKZ expression through Stat1 pathway, indicating that PKZ is a novel IFN stimulated gene. On the contrary, the suppressor of cytokine signaling 1 (SOCS-1), an inhibitor of the IFN signaling pathways, can significantly suppress the expression of *PKZ* (41).

Consistently, *PKZ* and *PKR* promoters contain IFN-stimulated response element (ISRE) that is required for their expression induced by IFN (42). There is at least one typical ISRE within *PKZ* promoter (one ISRE in *CiPKZ*, two ISREs in *CaPKZ*) (22, 43), which is significantly induced by poly(I:C) and IFN (22). Also, IRF3 and IRF7 can significantly activate *CiPKZ* promoter, but cannot effectively activate the truncated mutant *CiPKZ-nISRE-pro* that lacked ISRE (43), highlighting the relevance of PKZ during IFN-mediated antiviral response.

### Function as an Antiviral Effector

As a typical ISG encoding a novel eIF2α kinase, PKZ might inhibit viral replication, like PKR, through phosphorylating eIF2α, and thus resulting in inhibition of the synthesis of viral proteins. *In vitro* assays have shown that overexpression of PKZ significantly inhibit GCRV replication, while knockdown of this protein makes fish cells more vulnerable to virus infection. It is no doubt that PKZ can bind and phosphorylate eIF2α, and upon GCRV infection, the expression level of PKZ is consistent with the relatively level of eIF2α phosphorylation and the virus titer (22). Despite that PKR and PKZ are simultaneously induced during viral infection, fish PKR and PKZ form homodimers, but not heterodimers, to phosphorylate eIF2α independently, indicating fish PKZ and PKR play a non-redundant but cooperative role in IFN antiviral response.

In addition, PKZ might function as an antiviral effector by facilitating cell apoptosis. Overexpression of wild-type *Ci*PKZ (PKZ-wt) in CIK cells results in a striking decrease of cell viability rate. When PKZ-deficient cells were transfected with PKZ-wt rather than mutant PKZ-K198R, a significant increase of apoptotic cell number is observed. Also, this apoptosis is related to the eIF2α phosphorylation level (44). Due to host cells undergo apoptosis in response to virus infection and the induction of apoptosis is an antiviral innate immune mechanism (45, 46), it can be deduced that fish PKZ is indeed an antiviral effector. A question is how PKZ is activated during virus infection. It is noted that Z-DNA binding is indispensable to the regulation of PKZ activity. The enzyme missing the Zα domain (PKZΔN) is less effective than wild-type PKZ at inhibiting luciferase activity (20). As for *Dr*PKZ, deletion of the N-terminus leads to weakening the kinase activity compared with the wild-type (17).

### Function as a Cytosolic Sensor

It is interesting that two Z-DNA binding domain-containing proteins identified in mammals, DAI (ZBP1/DLM-1) and ADAR1, have been confirmed to be involved in IFN signaling. As a cytosolic sensor, DAI binds to cytosolic dsDNA initiating innate immune signal by activation of NF-κB and upregulation of type I IFN (47). In mammals, in addition to being an antiviral effector, PKR also acts as a dsRNA sensor detecting virus infection in the cytoplasm. Since fish PKZ can bind to Z-DNA or Z-RNA with high affinity, it is reasonable to infer that PKZ is a new sensor for Z-DNA/RNA in the cytoplasm (22). A recent study has shown that PKZ can trigger immune responses via IRF3- or ISGF3-like mediated pathways in fish (15). On account of the fact that DAI has not been found in the fish genome, perhaps PKZ is a kind of compensator for the lack of DAI, functioning as a cytosolic DNA sensor to trigger innate antiviral immune response. Nevertheless, a lot of questions need to be addressed. What’s the manner of PKZ recognizes DNA? What’s the relationship between PKZ and the candidate substrates? What’re the biological significances for PKZ-triggered innate immune response?

## Conclusion

In mammals, there are four members of eIF2α kinase family, and PKZ is the fifth member found in this family. Like PKR, PKZ inhibits viral replication through a similar mechanism to phosphorylate eIF2α. PKR has N-terminal dsRBDs, which enables PKR as a cytosolic receptor to recognize dsRNA generated during virus infection. Similarly, fish PKZ might function as a Z-DNA/Z-RNA sensor, to capture nucleic acid molecules and subsequently activate innate immune response. Despite of these findings, what is the source of Z-DNA/Z-RNA in the cytoplasm? What is the source of negative supercoiling in the cytoplasm? Given that Z-DNA is a transcription-dependent structure, it can be stabilized by the negative supercoiling generated by a moving RNA polymerase as it plows through the DNA double helix *in vivo*. We also do not known why PKZ is exclusively in fish genome. It is likely that the coexistence of PKR and PKZ is especially important to taxonomically lower fish species that live in a complex water environment, since harboring distinct N-terminal domains enable fish PKR and PKZ to recognize different viral nucleic acids, which makes the fish innate immune system more effective by broadening their ability to sense viruses and to defense against viral infection cooperatively.

## Author Contributions

CW created the figure and drafted the manuscript under the guidance of YZ and CH. YZ and CH edited and revised the drafts, respectively. All authors approved the final version.

## Conflict of Interest

The authors declare that the research was conducted in the absence of any commercial or financial relationships that could be construed as a potential conflict of interest.

## Abbreviations

ADAR: double-stranded RNA adenosine deaminase
*Ca*: *Carassius auratus*
*Ci*: *Ctenopharyngodon idellus*
DAI: DNA-dependent activator of interferon regulatory factors
*Dr*: *Danio rerio*
dsRBD: dsRNA binding domain
eIF2 α: the α subunit of eukaryotic translation initiation factor 2
IFN: interferon
IRF3: interferon transcription factor 3
ISGF3: interferon-stimulated gene factor 3
PKR: double-stranded RNA-dependent protein kinase
PKZ: protein kinase containing Z-DNA binding domains
*Ss*: *Salmo salar*
Z α: Z-DNA binding domain.
